# Construction and validation of a competency model for hospital operation assistant of public hospitals in China: a cross‑sectional study

**DOI:** 10.1186/s12913-023-10139-w

**Published:** 2023-10-23

**Authors:** Jia Gao, Meirong Tian, Jun Liu, Jingjing Chen, Lei Zhang, Xiaodong Wang, Ling Yan, Qiwang Liu, Jin Wen

**Affiliations:** 1 Institute of Hospital Management, West China Hospital, Sichuan University, Chengdu, Sichuan Province 610041 China; 2 Operation Management Department, West China Hospital, Sichuan University, Chengdu, Sichuan Province 610041 China

**Keywords:** Public hospital, Hospital operation assistant, Competency model

## Abstract

**Background:**

Hospital operation assistant (HOA) plays an important role in promoting the operation effectiveness and efficiency of hospital. China, as a resource-poor country, urgently needs to train HOA talent. The purpose of this study is to construct and validate a competency model for HOA, which can be used as a tool to select and train HOAs.

**Methods:**

Basic competency items were first constructed through literature review combined with the job analysis of HOA. Then, a questionnaire survey conducted on more than 300 hospital operation management-related staff was used to assess the importance of competency items. Exploratory factor analysis, structural equation model and second-order confirmatory factor analysis were used to construct and validate the competency model of HOA.

**Results:**

A total of 23 items were identified as critical to HOA capability, which were further divided into three factors: professional skills, professional knowledge and personality traits. The structural equation model showed that the standardized path coefficients of the three factors were 0.86, 0.82 and 0.98. The competency model passed strict fitting effect tests in several aspects, including root-mean-square error of approximation (RMSEA) = 0.077 (< 0.080), standardized root mean square residual (SRMR) = 0.062 (< 0.080), comparative fit index (CFI) = 0.927 (> 0.900) and Tucker-Lewis index (TLI) = 0.918 (> 0.900), which showed that the fitting validity of the model was ideal. The composite reliability (CR), average variance extracted (AVE) and correlation coefficients of all factors were within the standard range, which showed that the construction validity and discrimination validity of the model were ideal.

**Conclusion:**

Our study indicates that the competency model of HOA is an instrument with appropriate fit validity, construct validity and discriminant validity, which can provide criteria for selecting and training HOAs.

**Supplementary Information:**

The online version contains supplementary material available at 10.1186/s12913-023-10139-w.

## Introduction

A high-quality development system for public hospitals should be built, according to *Opinions of the General Office of the State Council on Promoting the High-Quality Development of Public Hospitals* released by the General Office of the State Council of the People’s Republic of China in 2021 [1]. This notice called for public hospitals in China to make three changes, namely, the development mode changed from scale expansion to quality and efficiency enhancement, the operation mode changed from extensive management to delicate management, and the resource allocation changed from focusing on material factors to focusing more on talent and technology factors. This poses a great challenge to the operation management of public hospitals in China.

Hospital operation management (HOM) is a series of management activities such as the design, planning, organization, implementation, control and evaluation of the internal operation of hospitals, which focuses on comprehensive budget management and business process management, and takes total cost management and performance management as tools. It is a series of management means and methods for the reasonable allocation, efficient management and effective use of core resources in hospitals, such as human, financial, material and technology resources. With the promulgation of the *Guideline on Strengthening the Public Hospitals Operation Management* [2], HOM has become one of the core management activities of public hospitals in China. To improve the system and promote the development of HOM, a hospital operation assistant (HOA) system needs to be established.

The purpose of HOA is to assist the director of the clinical department in strengthening internal operations and promoting management innovation. The important duties of HOA are to allocate resources, analyze the operation status of clinical department and hospital, and evaluate performance of hospital economic management activities. Overall, HOA plays three major roles: “eyes” to monitor the operation of clinical departments, “bridges” between functional departments and clinical departments, and “bonds” to promote hospital reform and innovation [3–8]. It has been demonstrated that HOA plays a key role in the system of HOM [4], so more and more hospitals are beginning to reserve professional HOAs.

However, in the development of public hospitals, the HOAs face great challenges. On the one hand, HOAs need to balance and coordinate the relationship between hospital strategic objectives and clinical department interests, which challenges their management ability and coordination ability. On the other hand, HOAs need to have comprehensive management knowledge such as operation analysis knowledge, equipment management knowledge, human resource assessment knowledge, performance management knowledge, medical management knowledge and so on [5]. So, the number of qualified HOAs in China is far from sufficient, compared with the needs of the development of HOM and the strategic expectation of national health development. Therefore, selecting and training qualified HOAs are important to hospitals. However, how to select, train and evaluate a qualified HOA has perplexed many healthcare managers.

Identification of the competency of HOA plays a critical role [9]. Competency was first put forward by McClellan in 1973 and it was widely used in the field of human resource management successfully. Subsequent scholars have conducted in-depth research on its concept, enriched the theoretical basis and reached a consistent view, that is, competency refers to measurable characteristics of a person, including knowledge, skills, self-concept, traits and motivations [10–12]. Competency-based models have been widely developed to identify the abilities of hospital managers. Liang et al., for example, developed a competency model of directors of medical services to explore the way of developing and improving competency [13]. In the study of Sitong Wang et al., a nurse manager competency model of tertiary general hospitals was constructed to select nurse managers [14]. Zhanming Liang et al. established a health service management competency model to measure the core management competencies of health service managers [15].

However, the competencies underlying HOA have not received much attention. Only Xin Zheng et al. used structural equation model to study the competency of clinical management assistants in hospitals, and divided the competency items into three factors: personality traits, ability and thinking [16]. However, the indexes of fitting validity, convergence validity, and discriminant validity indicated that the fitting effect of the model still needed to improve continuously.

Therefore, establishing a competency model for HOA of public hospitals is expected, innovative and has practical application value. In this study, by introducing competency model theory, we aimed to build a quantifiable competency model for HOA based on the exploratory factor analysis method and structural equation model. We hope that this model can provide standards for selecting and training HOAs in public hospitals in China.

## Methods

### Instrument development

To identify and generate the competency items of HOA, we used two strategies: job analysis and literature review. Job analysis is a systematic empirical procedure for collecting and analyzing information about a job and it has been widely used in the field of human resource management [17, 18]. West China Hospital of Sichuan University is the first hospital in Chinese Mainland to implement the HOM system, and it has trained a number of mature HOAs. Therefore, we conducted unstructured interviews with the HOAs of West China Hospital of Sichuan University to obtain the main responsibilities of HOA. The literature review was performed by searching the Web of Science, China National Knowledge Infrastructure, and Wanfang Database. Then we sort out the competency literature related to hospital management and HOM to extracted the competency items of the HOA.

### Questionnaire and participants

A cross-sectional survey design was used in this study. We sent an online questionnaire to 373 participants who participated in the Hospital Operation Assistant Training Course organized by West China Hospital of Sichuan University. Most of these participants were the directors or HOAs from the hospital operation management department. Of course, some public hospitals in China have not yet established dedicated hospital operation management departments, but these Chinese hospital directors have realized the significance of HOM and promoted relevant work by assigning the hospital operation management functions to other departments such as performance management departments, human resources departments, etc. [19]. Therefore, some participants in the training course were the directors or management cadres who came from the hospital human resource management department, performance management department, and finance department and so on. The questionnaire contained three sections: hospital information, personal information, and the importance ratings for competency items. All items were rated using a 5-point Likert scale, ranging from “very unimportant” (1 point) to “very important” (5 points) (Additional file 1).

### Statistical analysis

Descriptive statistical indicators were calculated to describe the characteristics of sample data. Continuous variables were described using mean and standard deviation, while categorical variables were analyzed using frequency, percentage and full-score proportion. Cronbach’s alpha (Cronbach’s α) coefficient, the Kaiser-Mayer-Olkin (KMO) sampling adequacy test and Bartlett’s sphericity test were used to test the reliability and validity of the questionnaire [20].

Exploratory factor analysis (EFA) was used [21]. Factors were extracted according to the principle that the initial eigenvalues were greater than 1.0 and the varimax rotation method was used to identify the role of each factor clearly [22, 23]. We used covariance-based structural equation model to analysis the relationship between the factors. The competency model path map was drawn and the standardized path coefficients were calculated [24]. Then, we used the second-order confirmatory factor analysis to validate the model’s fitting effect including fitting validity, convergent validity and discriminant validity. We used root-mean-square error of approximation (RMSEA < 0.080), standardized root mean square residual (SRMR < 0.080), comparative fit index (CFI > 0.90) and Tucker-Lewis index (TLI > 0.90) to test the fitting validity [25–27]. The standardized factor load, composite reliability (CR > 0.800) and average variance extracted (AVE > 0.500) were used to verify the convergence validity [28, 29]. The relationship between the square root of AVE and the correlation coefficient between factors was used to identify the discriminant validity. If the square root of AVE is greater than the correlation coefficient between factors, the discriminant validity is good [28–30]. All the data were analyzed by SPSS 19.0 and AMOS 23.0.

### Ethical issues

The study did not address privacy or ethical issues and all methods were carried out in accordance with relevant guidelines and regulations. First, we provided all participants with written survey information and promised that the survey results would only be used for scientific research. So, all informed consent was obtained from all subjects. Second, an anonymous survey was adopted. Finally, participants had the right to refuse to answer questions or withdraw from the study at any time.

## Results

### Competency items of the HOA

After job analysis, we concluded the main responsibilities of the HOA as follows.

1. Under the guidance of the clinical department director and the hospital operation management department director, HOA needs to organically combine the hospital’s macro-strategy with the department’s development strategy, and takes the initiative to coordinate, communicate and interact between different departments.
2. HOA assists the clinical department director in managing, so that the clinical department can implement various policies of the hospital.
3. HOA needs to complete the routine operation analysis of the HOM on time, such as the maintenance and update of the basic data of clinical departments, the operation analysis of clinical departments, the usage analysis of key medical equipment and so on.
4. HOA needs to complete the evaluation and demonstration of the resource in clinical department on time, such as human resource evaluation, clinical equipment investment benefit analysis, cost analysis and so on.
5. HOA needs to communicate with all departments of the hospital actively.
6. HOA needs to complete other special work assigned by hospital.

From the results of job analysis, HOA needs lots of professional knowledge, skills, and experience. Ulteriorly, based on the job analysis results and literature review, we screened out 23 competency items of HOA preliminarily and made a summary definition of these items (Table 1).

**Table 1:** The competency items of HOA and summary definition

**Competency item**	**Summary definition**
Data processing and analysis	Clean and analyze hospital data to explore the potential value of the data deeply
Language and writing ability	The language expression ability and the writing ability
Structured thinking	When facing work problems or challenges, one can think about the problem from multiple perspectives, analyze the reasons deeply, and develop systematic solutions
Interpersonal communication	Create a good communication atmosphere, adopt effective communication methods, express accurately, and be able to handle various verbal conflicts and contradictions
Innovation ability	Be good at discovering new problems, adopting new methods, and generating new results
Teamwork and cooperation	Possess teamwork spirit and cooperate with others actively
Adaptability	Strong adaptability to various scenarios
Self-improvement	Having intense motivator to improve one’s abilities continuously and pursue self-worth
Financial and economic knowledge	Master frequently-used theoretical knowledge and methods related to finance and economics, such as sunk cost, time value and cost-volume-profit analysis methods and so on
Management theoretical knowledge	Master theoretical knowledge related to management, such as management functions and principles, organizational management theory and so on
Medical knowledge	Master medical-related theoretical knowledge, such as the pathogenesis and treatment methods of common diseases and so on
Statistical knowledge	Master theoretical knowledge and methods related to statistics, such as statistical description, statistical inference, regression analysis and so on
Social science knowledge	Master theoretical knowledge related to social sciences, such as sociology, psychology, political science and so on
Anti-pressure ability	Able to withstand pressure and possess strong self-regulation ability
Open-minded	Willing to consider new ideas; unprejudiced
Empathy	Able to understand others’ feelings and experience
Self-evaluation	Evaluate oneself correctly and objectively
Sense of responsibility	An awareness of your obligations and having the driving force for completing your own tasks consciously
Professional identity	Sincerely identify with the value of the work you are engaged in
Organizational loyalty	Proud of the organization and willing to actively participate in various activities related to its development
Organizational identity	Organizational recognition of one’s job and personal values
Medical experience	Have clinical work experience
Management experience	Having management work experience

### Characteristics of the respondents

We collected a total of 318 questionnaires from 153 hospitals in 25 provinces of China. The response rate was 85.25% (318/373). Nine were excluded because of incomplete answers, which left 309 useable questionnaires. All the respondents were aged 23 to 57 years, among whom 47.57% were aged 31 to 40 years, 70.87% were females, 55.02% had obtained a bachelor’s degree, 41.74% had obtained a master’s degree or a doctor’s degree, 86.41% were from tertiary hospitals, and 58.90% were directly engaged in the work of HOM (Table 2).

**Table 2:** Characteristics of the respondents

**Characteristics**	**Frequency (*n* = 309)**	**Percentage (%)**
Gender		
Female	219	70.87
Male	90	29.13
Age (year)		
23–30	75	24.27
31–40	147	47.57
41–49	69	22.33
50–57	18	5.83
Education level		
Junior college and below	10	3.24
Bachelor’s degree	170	55.02
Master’s degree	122	39.48
Doctoral degree	7	2.26
Hospital level		
Tertiary hospital	267	86.41
Secondary hospital	24	7.77
Others	18	5.82
Responsibilities		
HOM	182	58.90
Financial Management	32	10.36
Performance Management	23	7.44
Human Resources Management	12	3.88
Others	60	19.42

### Descriptive statistics of the HOA competency items

The mean score, maximum score, and minimum score of the 23 competency items of HOA were 4.62,4.82 and 4.22, respectively. The standard deviation of each item was less than 1.00. The highest full-score proportion of the items was 83.17%, and the lowest was 40.13% (Table 3).

**Table 3:** Importance scores of 23 competency items

**No.**	**Competency item**	**Means**	**SD**	**Full-score proportion (%)**
1	Data processing and analysis	4.82	0.45	83.17
2	Structured thinking	4.81	0.42	82.52
3	Interpersonal communication	4.81	0.41	81.55
4	Self-improvement	4.81	0.40	80.91
5	Teamwork and cooperation	4.80	0.42	80.91
6	Adaptability	4.72	0.53	74.76
7	Innovation ability	4.71	0.55	74.76
8	Professional identity	4.71	0.46	70.87
9	Open-minded	4.69	0.51	70.55
10	Language and writing ability	4.68	0.50	69.90
11	Anti-pressure ability	4.68	0.54	72.17
12	Organizational loyalty	4.68	0.51	69.58
13	Self-evaluation	4.64	0.53	66.02
14	Management theoretical knowledge	4.62	0.56	65.37
15	Sense of responsibility	4.61	0.57	64.72
16	Organizational identity	4.58	0.54	60.84
17	Empathy	4.57	0.56	60.84
18	Statistical knowledge	4.51	0.58	54.69
19	Financial and economic knowledge	4.49	0.60	54.37
20	Medical knowledge	4.41	0.65	49.19
21	Management experience	4.40	0.67	50.16
22	Medical experience	4.26	0.78	43.69
23	Social science knowledge	4.22	0.75	40.13

### Reliability and validity test

The Cronbach’s α coefficient of the competency questionnaire was 0.957, which was greater than 0.900, indicating that the questionnaire items had good internal consistency. The KMO value was 0.964, which was greater than 0.900, indicating that the structural validity of the questionnaire items was very good. Bartlett’s sphericity test showed that the χ^2^ value was 7067.029 (*P* < 0.001), indicating that there was a strong correlation between competency items, which confirmed the appropriateness of the factor analysis technique.

### Exploratory factor analysis

Three principal factors were extracted according to the principle that the initial eigenvalues were greater than 1.0. These factors cumulatively explained 67.60% of the total variance, which was basically acceptable. After varimax rotation, all the items had factor loadings greater than 0.50, so they were all retained (Table 4).

**Table 4:** The factor loading matrix after rotation

**Items**	**Factor 1**	**Factor 2**	**Factor 3**
Data processing and analysis	0.638		
Language and writing ability	0.684		
Structured thinking	0.775		
Interpersonal communication	0.784		
Innovation ability	0.707		
Teamwork and cooperation	0.763		
Adaptability	0.616		
Self-improvement	0.760		
Financial and economic knowledge		0.759	
Management theoretical knowledge		0.586	
Medical knowledge		0.797	
Statistical knowledge		0.716	
Social science knowledge		0.790	
Anti-pressure ability			0.741
Open-minded			0.774
Empathy			0.687
Self-evaluation			0.664
Sense of responsibility			0.642
Professional identity			0.671
Organizational identity			0.616
Organizational loyalty			0.660
Medical experience		0.759	
Management experience		0.746	

Factor 1 included eight competency items (data processing and analysis, language and writing ability, structured thinking, interpersonal communication, innovation ability, teamwork and cooperation, adaptability and self-improvement), which reflected the basic professional skills that the HOA should have, so it was named “professional skills”. Considering that the contents of 7 items (financial and economic knowledge, management theoretical knowledge, medical knowledge, statistical knowledge, social science knowledge, medical experience and management experience) under factor 2 reflected the professional knowledge of the HOA, so we named it “professional knowledge”. The 8 items (anti-pressure ability, open-minded, empathy, self-evaluation, sense of responsibility, professional identity, organizational identity and organizational loyalty) under factor 3 reflected the psychological quality and professional sense of the HOA, so we named factor 3 “personality traits”.

### Structural equation model

**Fig. 1:**
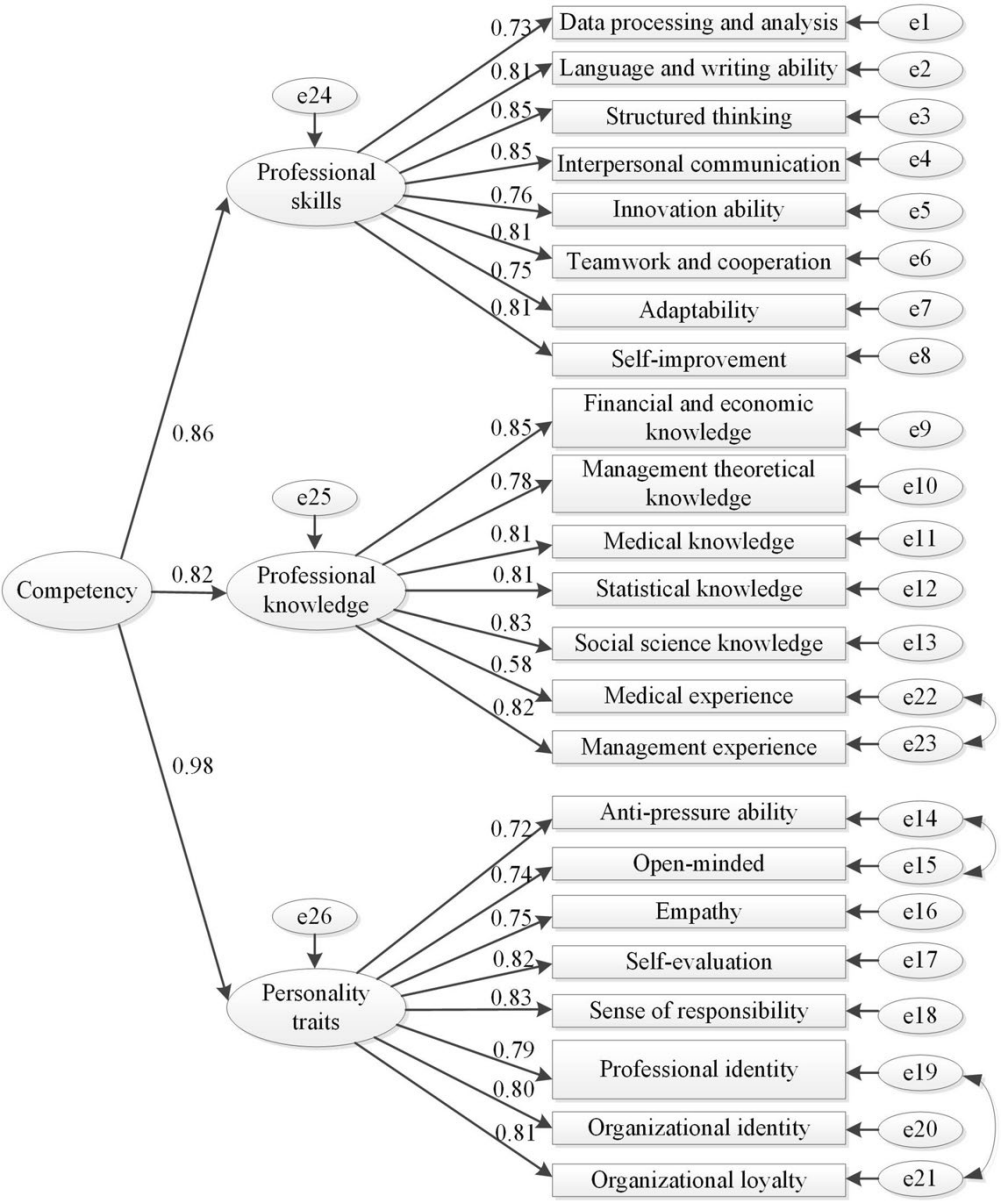
The final path map of the competency model for HOA. Second-order potential variable, first-order potential variables and manifest variables are connected by significant paths. The numbers on the straight arrows indicate the standardized path coefficients

According to the results of factor analysis, taking the competency of the HOA as the second-order potential variable, “professional skills”, “professional knowledge” and “personality traits” as the first-order potential variables, and the 23 competency items as the observation variables of the three factors, the competency model path map was drawn and the standardized path coefficients were calculated by using AMOS 23.0. The standardized path coefficient of professional skills, professional knowledge and personality traits were 0.86, 0.82 and 0.98 respectively. The competency items’ standardized path coefficients were all greater than 0.50. All these standardized path coefficients were significant (*p* < 0.001). The final path map of the competency model with 3 factors and 23 items is illustrated in Fig. 1.

### Validation of the competency model

The overall model fitness was acceptable, where the RMSEA (0.077) and SRMR (0.062) were both less than 0.08, and the CFI (0.927) and TLI (0.918) indexes were both greater than 0.90. According to the results that all the standardized path coefficients were greater than 0.50 (*P* < 0.001), the CRs of all dimensions were all greater than 0.800, and the AVEs were above 0.500, the model had good convergence validity (Table 5). The results showed the correlation coefficients between the three factors were all less than or very close to each square root of AVE, indicating the competency model of HOA had good discriminant validity (Table 6).

**Table 5:** The convergence validity of the competency model

	**CR**	**AVE**
Competency	0.910	0.772
Professional skills	0.933	0.636
Professional knowledge	0.919	0.620
Personality traits	0.927	0.614

**Table 6:** The discriminant validity of the competency model

**Factors**	**Professional skills**	**Professional knowledge**	**Personality traits**
Professional skills	**0.797**		
Professional knowledge	0.780	**0.787**	
Personality traits	0.812	0.706	**0.783**

The diagonal data are the square root of the AVE

## Discussion

In this study, we built a competency model constituted by three factors (professional skills, professional knowledge and personality traits) and 23 items for HOA. The greater the standardized path coefficient of the structural equation model, the larger the impact of the indicator on competency. This research showed that the standardized path coefficient of personality traits was largest, which was 0.98, indicating that this index had the greatest impact on competency. This result is consistent with relevant research [16].

All the means of the 23 competency items were above 4.00, indicating that the peers of HOM believed that these 23 items were important for HOAs. The top five items in terms of importance were data processing and analysis, structure thinking, interpersonal communication, self-improvement and teamwork and cooperation. The item with the highest score was data processing and analysis, which indicated that it was most important for the HOAs to master the ability of cleaning and analyzing data to find abnormal operation status of hospitals in a timely manner. This is consistent with the HOA’s main job responsibilities which is completing the routine operation analysis of hospital [5]. Structured thinking ability came next, indicating that HOAs should not only be able to analyze the overall problem horizontally, but also be able to conduct in-depth analysis vertically so that they can analyze the problem comprehensively and clearly. Interpersonal communication, self-improvement, teamwork and cooperation ranked third to fifth respectively, indicating that HOAs needed to promote the implementation of hospital management through good communication and cooperation, and should be able to summarize and rethink profoundly in time to improve the management effect continuously.

These items that we have constructed for HOA competency model are consistent with the T-shaped model which requires managers to have a broad range of knowledge, cross-border comprehensive abilities and qualities (“—” type talents), and profound professional knowledge (“|” type talents) [31, 32]. Likewise, horizontally, HOAs need to possess a broad range of knowledge (such as statistical knowledge, management theoretical knowledge, financial and economic knowledge, medical knowledge, social science knowledge and so on), comprehensive abilities and qualities (such as language and writing ability, structured thinking, interpersonal communication and so on). Vertically, deep professional knowledge and skills are required (such as data processing and analysis).

There are also important practical significances in selecting and training HOAs. To select the suitable personnel, according to this model, HR personnel can measure the management knowledge and management skills of candidates by setting up questions about hospital financial analysis, hospital management knowledge, hospital management tools and so on. Since the personality traits’ standardized path coefficient (0.98) is the largest and personality traits are relatively stable [33], it is important to test candidates in advance through the discussion of the leaderless group, stress interviews, and self-assessment scale tests.

To improve the comprehensive competences of HOAs, a training mode combining theoretical teaching and practical training can be built from the modules of expertise, basic skills and professional quality. First, in terms of expertise, basic theoretical knowledge of management, hospital financial management, hospital information management, and health statistics can be fit into the training course. Second, for the training of professional skills, project practice, data analysis and processing, speech and expression training, communication training, thinking logic training and other skill courses can be carried out. Finally, for the shaping of personality traits, on-site visits, experience exchange, scenario simulation, and action learning can be set up to improve the position awareness and professional quality of participants.

### Limitations

Our study had several limitations. Although the samples for the study were extensive, covering 153 hospitals in 25 provinces of China, no distinction was made between different levels or types of public hospitals. In the future, it is necessary to further expand the scope and the number of survey samples for deep exploration. Another limitation is that our sampling method was not strictly random and the answers could be biased. In addition, further research needs to be conducted on how to accurately measure each competency item.

## Conclusion

Based on the literature review, job analysis and a questionnaire survey, a competency model of HOA was established by adopting factor analysis and structural equation model. The fitting effect of the model was ideal. It enriched the research in the field of HOA’s competency and can provide criteria for selecting and training HOAs.

### Supplementary Information

**Additional file 1.** Questionnaire on the Competency of Hospital Operation Assistants Statement.

## Data Availability

The datasets used and/or analysed during the current study are available from the corresponding author on reasonable request.

## Abbreviations

HOM: Hospital Operation Management
HOA: Hospital Operation Assistant
KMO: Kaiser-Mayer-Olkin
EFA: Exploratory factor analysis
RMSEA: Root-mean-square Error of Approximation
SRMR: Standardized Root Mean Square Residual
CFI: Comparative Fit Index
TLI: Tucker-Lewis Index
CR: Composite Reliability
AVE: Average Variance Extracted
