# Supplementary material for: Construction and validation of a competency model for hospital operation assistant of public hospitals in China: a cross‑sectional study
Source: BMC Health Serv Res. 2023 Oct 23;23:1138. doi: 10.1186/s12913-023-10139-w (PMC10594754; doi:10.1186/s12913-023-10139-w)
Supplement: Supplementary file 1 — Additional file 1. Questionnaire on the Competency of Hospital Operation Assistants Statement. [file 12913_2023_10139_MOESM1_ESM.pdf]

Questionnaire on the Competency of Hospital Operation Assistants

Statement

Dear colleagues:

Thanks for your attention. In order to study the influencing factors of the competency of hospital operation assistant, we have developed a questionnaire on the competency of hospital operation assistants. This questionnaire is anonymous and does not involve personal privacy such as your name and phone number and so on. The survey data will only be used for scientific research. If possible, we invite you to fill out this questionnaire. Of course, you can refuse to answer questions without any worries or withdraw from the study at any time.

Thanks for your support!

Hospital information

1. Name of your hospital: ________________________________

2. Province:__________________ , City:____________________

3. please choose your hospital level:

A. Third-level Grade-A

B. Third-level Grade-B

C. Second-level Grade-A

D. Second-level Grade-B

E. Others

4. please choose your hospital type

A. general hospital

B. Specialized Hospital

Personal information

5. please choose your gender

A. male

B. female

6. Please fill in your age: __________________ (year)

7. Please choose your highest educational background

A. Doctoral degree

B. Master's degree

C. Bachelor's degree

D. Junior college and below

8. Please choose your working department

A. Human Resources department

B. Financial Management department

C. hospital operation management department

D. Performance Management department

E. Others

9. Please fill in your position: _________

10. Please write down your years of experience in this job position: ___________

Research on the importance of Competency items of Hospital Operations Assistants

11.Based on your management experience, please rate the importance of the competency items that hospital operation assistants should possess. 1 point represents "least important", 2 points represents "not very important", 3 points represents "generally important", 4 points represents "relatively important", and 5 points represents "very important"

**Competency item**	1	2	3	4	5
Data processing and analysis	○	○	○	○	○
Language and writing ability	○	○	○	○	○
Structured thinking	○	○	○	○	○
Interpersonal communication	○	○	○	○	○
Innovation ability	○	○	○	○	○
Teamwork and cooperation	○	○	○	○	○
Adaptability	○	○	○	○	○
Self-improvement	○	○	○	○	○
Financial and economic knowledge	○	○	○	○	○
Management theoretical knowledge	○	○	○	○	○
Medical knowledge	○	○	○	○	○
Statistical knowledge	○	○	○	○	○
Social science knowledge	○	○	○	○	○
Anti-pressure ability	○	○	○	○	○
Open-minded	○	○	○	○	○
Empathy	○	○	○	○	○
Self-evaluation	○	○	○	○	○

Sense of responsibility	○	○	○	○	○
Professional identity	○	○	○	○	○
Organizational loyalty	○	○	○	○	○
Organizational identity	○	○	○	○	○
Medical experience	○	○	○	○	○
Management experience	○	○	○	○	○

Thanks for your substantial support again!
